# Effect of protective lung ventilation on pulmonary complications after laparoscopic surgery: a meta-analysis of randomized controlled trials

**DOI:** 10.3389/fmed.2023.1171760

**Published:** 2023-05-25

**Authors:** Menglin Sun, Ruolin Jia, Lijuan Wang, Daqi Sun, Mingqian Wei, Tao Wang, Lihua Jiang, Yuxia Wang, Bo Yang

**Affiliations:** ^1^Department of Anesthesiology, Third Affiliated Hospital of Zhengzhou University, Zhengzhou, China; ^2^Department of Obstetrics and Gynecology, Third Affiliated Hospital of Zhengzhou University, Zhengzhou, China

**Keywords:** protective lung ventilation, small tidal volume, moderate PEEP, laparoscopic surgery, pulmonary complications, meta-analysis

## Abstract

**Introduction:**

Compared with traditional open surgery, laparoscopic surgery is widely used in surgery, with the advantages of being minimally invasive, having good cosmetic effects, and having short hospital stays, but in laparoscopic surgery, pneumoperitoneum and the Trendelenburg position can cause complications, such as atelectasis. Recently, several studies have shown that protective lung ventilation strategies are protective for abdominal surgery, reducing the incidence of postoperative pulmonary complications (PPCs). Ventilator-associated lung injury can be reduced by protective lung ventilation, which includes microtidal volume (4–8 mL/kg) ventilation and positive end-expiratory pressure (PEEP). Therefore, we used randomized, controlled trials (RCTs) to assess the results on this topic, and RCTs were used for meta-analysis to further evaluate the effect of protective lung ventilation on pulmonary complications in patients undergoing laparoscopic surgery.

**Methods:**

In this meta-analysis, we searched the relevant literature contained in six major databases—CNKI, CBM, Wanfang Medical, Cochrane, PubMed, and Web of Science—from their inception to October 15, 2022. After screening the eligible literature, a randomized, controlled method was used to compare the occurrence of postoperative pulmonary complications when a protective lung ventilation strategy and conventional lung ventilation strategy were applied to laparoscopic surgery. After statistical analysis, the results were verified to be statistically significant.

**Results:**

Twenty-three trials were included. Patients receiving protective lung ventilation were 1.17 times less likely to develop pulmonary complications after surgery than those receiving conventional lung ventilation (hazard ratio [RR] 0.18, 95% confidence interval [CI] 1.13–1.22; *I*^2^ = 0%). When tested for bias (*P* = 0.36), the result was statistically significant. Patients with protective lung ventilation were less likely to develop pulmonary complications after laparoscopic surgery.

**Conclusion:**

Compared with conventional mechanical ventilation, protective lung ventilation reduces the incidence of postoperative pulmonary complications. For patients undergoing laparoscopic surgery, we suggest the use of protective lung ventilation, which is effective in reducing the incidence of lung injury and pulmonary infection. Implementation of a low tidal volume plus moderate positive end-expiratory pressure strategy reduces the risk of postoperative pulmonary complications.

## Introduction

Laparoscopic surgery is a technique that uses a laparoscope in the abdominal cavity to monitor and guide surgery from outside the abdomen to complete the exploration of diseased tissue, haemostasis, electrocoagulation, suturing and other operations. Laparoscopic surgery is widely used because of its low rate of bleeding, low postoperative pain (1), fast recovery, and short hospital stays. Compared with traditional open surgery, laparoscopy is widely used in surgery with the advantages of minimal invasiveness, good cosmetic effects and short hospital stays. However, during laparoscopic surgery, pneumoperitoneum and Trendelenburg positions can cause postoperative pulmonary complications (PPCs), such as atelectasis (2), resulting in severe perioperative respiratory dysfunction. Studies have shown that the incidence of PPCs after general surgery is 5%, while the incidence of PPCs after abdominal surgery is between 12% and 58% (1).

Mechanical ventilation is a routine surgical form of ventilation that used to use high tidal volume ventilation (10 to 15 mL/kg) to prevent hypoxaemia and atelectasis. However, experiments have shown that mechanical ventilation under high tidal volume ventilation conditions can cause alveolar hyperexpansion, worsen lung injury, and cause ventilator-related lung injury (3). Recently, several studies have shown that certain lung ventilation strategies are protective for abdominal surgery, reducing the incidence of PPCs (4, 5).

Protective lung ventilation minimizes lung injury and circulatory suppression due to mechanical ventilation while improving hypoxaemia. Intraoperative protective ventilation strategies can maintain alveolar dilation, reduce alveolar collapse or over dilation, and decrease the incidence of atelectasis. The core components of protective pulmonary ventilation include small tidal volume ventilation [Vt 6–8 mL/kg (6, 7)], moderate positive end-expiratory pressure [PEEP 5–10 cm H_2_O (6, 8)], and pulmonary recruitment.

A high tidal volume can be used to reopen an area of the lung where the end of the expiratory has collapsed and repair arterial oxygenation injury, but it is considered safe only for short periods of mechanical ventilation. Appropriate positive end-expiratory pressure can be effective in preventing PPCs. High PEEP can promote alveolar hyperexpansion, pulmonary vascular resistance can increase accordingly (1), and ventilatory blood flow ratio imbalance can impair haemodynamics, causing postoperative pulmonary complications, and the ideal PEEP value is currently unclear. However, all relevant studies have recommended small tidal volumes, and there is clear evidence that protective lung ventilation in patients with acute lung injury and acute respiratory distress syndrome is effective in reducing morbidity and mortality (8). Nevertheless, the effect is not obvious in the general patient population, and there is a lack of strong evidence and clear mechanisms to prove that protective lung ventilation can be effective in reducing the occurrence of pulmonary complications when applied to laparoscopic surgery.

Therefore, we used randomized, controlled trials (RCTs) to assess the results on this topic and for meta-analysis to further assess the effects of protective lung ventilation (low tidal volume ventilation and PEEP) on pulmonary complications in laparoscopic surgery patients.

## Methods

### Search strategy

In this systematic review and meta-analysis, we submitted a registration for this study on the PROSPERO website and is currently being assessed. We followed the PRISMA (9) guidelines (PRISMA Checklist can be seen in Appendix 1) and collected articles from six Chinese and English literature databases—CNKI, Medical Wanfang, CBM, Cochrane, PubMed, and Web of Science—as well as relevant subject literature from the China Clinical Trial Registry through a literature search, without language restrictions. Randomized, controlled trials were searched for according to the corresponding keywords and extended terms in Chinese and English, and all relevant articles from the establishment of the database up to November 2022 were retrieved.

The complete detailed search string for PubMed was as follows: ((“Laparoscopes”[Mesh]) OR ((((((((((Peritoneoscope[Title/Abstract]) OR (Celioscope[Title/Abstract])) OR (Laparoscope[Title/Abstract])) OR (Laparoscopic surgery[Title/Abstract])) OR (Porous laparoscopy[Title/Abstract])) OR (Single-port laparoscopy[Title/Abstract])) OR (Transumbilical laparoscopy[Title/Abstract])) OR (Transumbilical single-port laparoscopy[Title/Abstract]))) AND ((“Pulmonary Ventilation”[Mesh]) OR (((((((((((((Ventilation, Pulmonary[Title/Abstract]) OR (Airflow, Respiratory[Title/Abstract])) OR (Airflow, Expiratory[Title/Abstract])) OR (Protective pulmonary ventilation[Title/Abstract])) OR (Protective ventilation[Title/Abstract])) OR (Pulmonary protective ventilation[Title/Abstract])) OR (Lung protective ventilation[Title/Abstract])) OR (Lung protective strategies[Title/Abstract])) OR (Lung-protective ventilation therapy[Title/Abstract])) OR (Pulmonary protective ventilation mode[Title/Abstract])) mechanical ventilation[Title/Abstract]))))AND(((randomized controlled Trial[Publication Type] OR (randomized[Title/Abstract])) OR (placebo[Title/Abstract])). The search strategies of other search engines can be seen in Appendix 2.

### Inclusion criteria and exclusion criteria

After completing the initial search of the literature, preliminary screening was performed by removing duplicate literature; excluding reviews, meta-analyses, systematic reviews, and literature with inconsistent research content by reading titles and abstracts; and selecting the literature that needed to be obtained in the original language by formulating inclusion and exclusion criteria and final evaluation indicators. The inclusion criteria were the following: (1) Study subjects: patients undergoing laparoscopic surgery; (2) Interventions: conventional lung ventilation strategies were in the control group and protective lung ventilation strategies used in the experimental group; (3) Outcome measures: at least one of the following: pulmonary complications: atelectasis, hypoxia, and hypoxaemia; (4) Study design: randomized, controlled trials (RCTs). Patients were randomly assigned to two groups, and the results of the two groups were compared. One group (experimental group) received an intervention with a protective lung ventilation strategy, while the other group (control group) received a conventional ventilation strategy. The two groups were compared for postoperative outcomes to determine the effectiveness of the intervention in the experimental group.

The exclusion criteria were: (1) repeatedly reported studies; (2) valid outcome measures not being obtained, e.g. atelectasis, hypoxia, and hypoxaemia; (3) additional measures added to the experimental group intervention; (4) the experimental design not matching in that protective lung ventilation strategies were used in the intervention group, and conventional pulmonary ventilation was used in the control group. Finally, by reading the original texts, the final relevant documents were obtained by eliminating the documents that did not meet the requirements.

In this review, we define:P as a patient who requires laparoscopic surgery; I means: the use of protective lung ventilation strategy as an intervention; C means: the control group uses the conventional lung ventilation strategy; O means: The outcomes of this meta-analysis are pulmonary complications; S means: the experimental design protocol is a fully randomized controlled trial. Pulmonary complications include: Pneumonia, Respiratory failure, Pulmonary embolism, Pulmonary embolism, Bronchopleural fistula, Pleural empyema. To investigate the effects of protective lung ventilation on pulmonary complications after laparoscopic surgery. Primary outcomes are: pulmonary infection, atelectasis; Secondary outcomes are: cough, lung injury, etc.

### Data extraction and quality analysis

We read the extracted data and further confirmed the relevant data extracted. The following data were extracted from each entry: first author, year of publication, group and number of participants, population characteristics (weight, sex, age), tidal volume and PEEP value in the experimental group (protective lung ventilation group) and control group (conventional lung ventilation group). The main evaluation indicators were pulmonary complications, such as lung infection and atelectasis, and the secondary indicators were cough, lung injury, etc. [PPCs are defined in Table 1 (10)].

**Table 1:** Standardized PPCs according to the european perioperative clinical outcome definitions.

**Pneumonia**	**Patient received antibiotics for a suspected respiratory infection and met one or more of the following criteria: new or changed sputum, new or changed lung opacities, fever, white blood cell count >12 × 10^9^·L^−1^**
Respiratory failure	Postoperative arterial oxygen partial pressure <8 kPa (60 mm Hg) on room air, an arterial oxygen partial pressure to oxygen fraction ratio <40 kPa (300 mm Hg) or arterial oxyhaemoglobin saturation measured with pulse oximetry <90% and requiring oxygen therapy
Pulmonary embolism	Lung opacification with a shift of the mediastinum, hilum or hemidiaphragm toward the affected area and compensatory overinflation in the adjacent non-atelectoic lung
Pulmonary embolism	Diagnosed by CT angiography without severity grading
Bronchopleural fistula	Diagnosed by flexible bronchoscopy, persistently requiring reoperation
Pleural empyema	Fever, white blood cell count >12 × 10^9^· L^−1^ and CT scan

The results of this study were dichotomous variables, and we calculated the relative risk (RR) using 95% confidence intervals. Heterogeneity was quantified by the *I*^2^ statistic. If the *I*^2^ value was >50%, the heterogeneity was considered significant. A bias test was also performed. All statistical analyses were processed using Review Manager software (RevMan, version 5.3) and Stata software, version 14.

RCT methods were used in this study. The studies were assessed for complete random allocation, allocation concealment, blinding of participants and staffs, data integrity, selective reporting of study results, and other sources of bias (small sample size, conflict of interest, unbalanced baseline), completed literature quality assessment, heterogeneity testing, and bias testing.

## Results

### Literatures search

**Figure 1:**
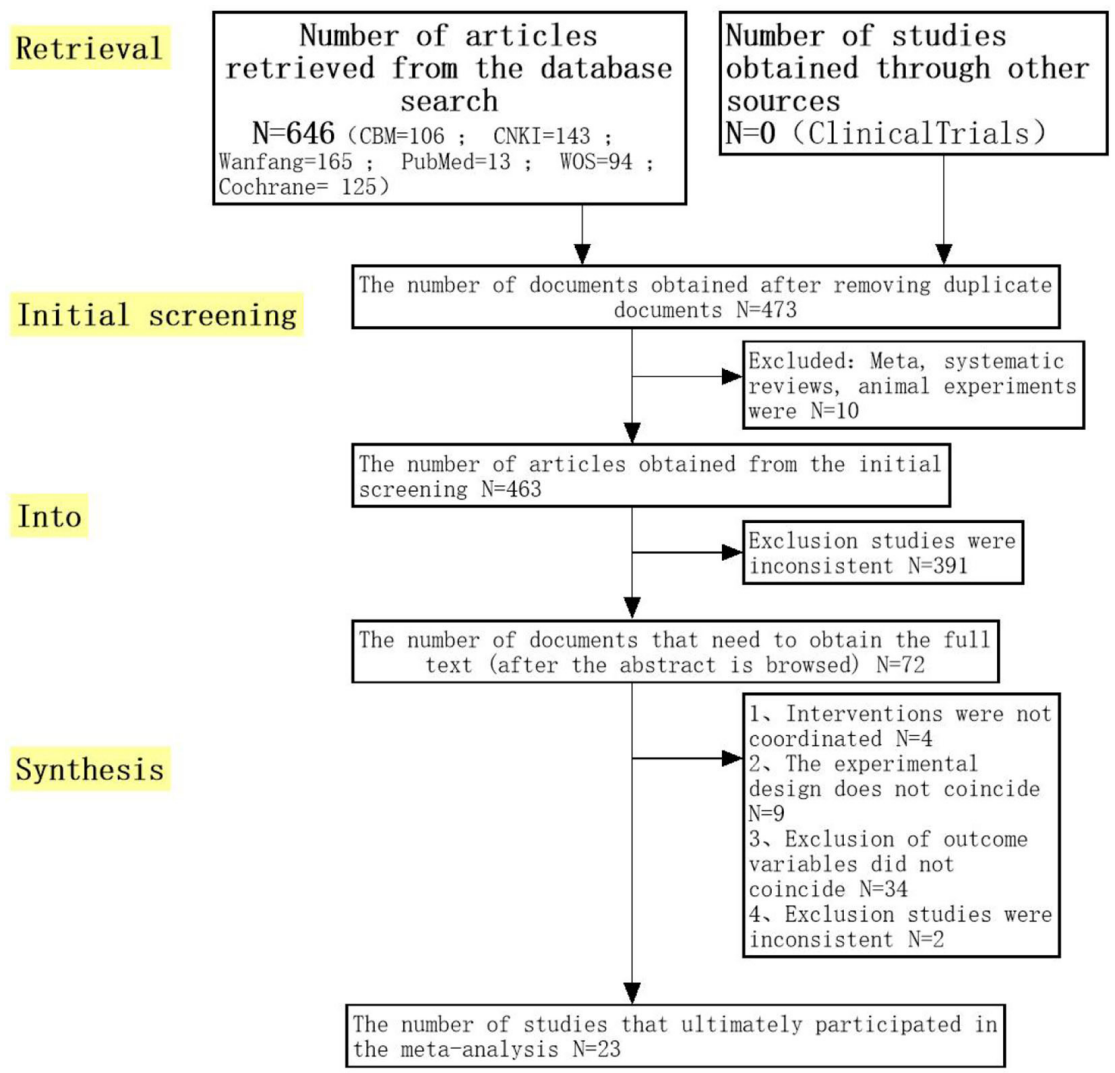
Selected randomized controlled trials (RCTs) for meta-analysis.

After a well-developed literature screening strategy, 646 articles were obtained. Two students read the titles and abstracts of these 646 articles alone, screened according to the inclusion and exclusion criteria formulated in advance, and summarized the articles screened by the two students together. By reading the title and summary, we excluded 173 duplicate articles. Then 574 articles were excluded due to non-compliance (Figure 1). The last 23 RCTs met the inclusion criteria for this meta-analysis.

The main characteristics of the 23 articles (1, 9, 11–31) of this study are shown in Table 2.

**Table 2:** Characteristics of randomized, controlled trials included in the meta-analysis.

	**Age**		**Gender (male/female)**		**Vt (ml/kg)**		**PEEP (cmH2O)**		**Number**		**Pulmonary complication (** ***N*** **)**	
	**P group**	**C group**	**P group**	**C group**	**P group**	**C group**	**P group**	**C group**	**P group**	**C group**	**P group**	**C group**
1	59.0 ± 9.0	55.0 ± 12.0	9/22	13/18	7	10	10		31	31	10	11
2	51.1 ± 8.9	50.3 ± 9.8	/	/	7	9	30		44	43	4	11
3	/	/	/	/	6–8	8–10	6		35	35	0	7
4	69.7 ± 5.8	70.8 ± 5.8	98/42	102/38	6	10	5		130	130	24	41
5	52.8 ± 16.5	57.4 ± 10.1	14/7	9/10	6	10	5		21	19	3	9
6	63.8 ± 9.7	68.2 ± 8.3	18/14	15/9	7	9	5		32	28	2	4
7	69.9 ± 6.3	68.6 ± 4.0	11/9	12/8	6	6	7	12	22	22	1	4
8	64.0 ± 6.0	62.0 ± 4.0	23/22	26/19	8	8	5		45	45	3	11
9	70.7 ± 7.1	71.1 ± 6.7	10/13	20/16	6–8	8–10	8–10		36	36	1	7
10	70.6 ± 9.3	70.2 ± 9.4	40/22	39/23	7	8–10	5		62	62	9	21
11	69.3 ± 3.0	70.2 ± 4.3	22/18	25/15	6	10	6		40	40	2	5
12	66.5 ± 8.3	66.1 ± 9.2	35/23	34/23	7	10	7		58	57	1	8
13	55.4 ± 10.7	56.0 ± 12.9	17/18	18/12	6–8	6–8	2		35	30	2	4
14	47.8 ± 12.0	50.0 ± 10.0	12/8	14/6	6	10	5		20	20	2	7
15	51.3 ± 10.3	54.4 ± 6.8	/	/	7	9	7		30	30	0	4
16	43.2 ± 7.3	43.2 ± 7.3	/	/	6	10	8–10		45	45	1	5
17	/	/	/	/	6	10	8–10		30	30	3	8
18	53.3 ± 7.3	52.5 ± 7.0	36/13	32/17	6	9	5		49	49	9	15
19	57.6 ± 5.0	56.9 ± 5.2	12/8	10/10	6	6	5		20	20	1	2
20	68.4 ± 4.0	69.1 ± 4.7	16/14	13/17	6	8	5		30	30	2	3
21	56.2 ± 4.3	57.5 ± 4.8	25/15	21/19	6–8	6–8	10	5	40	40	5	6
22	63.0 ± 6.0	63.0 ± 8.0	56/24	52/28	6	9	5		80	80	14	25
23	1.5 ± 0.5	1.7 ± 0.3	12/8	11/9	7	10	6		20	20	1	2

### Data analysis

**Figure 2:**
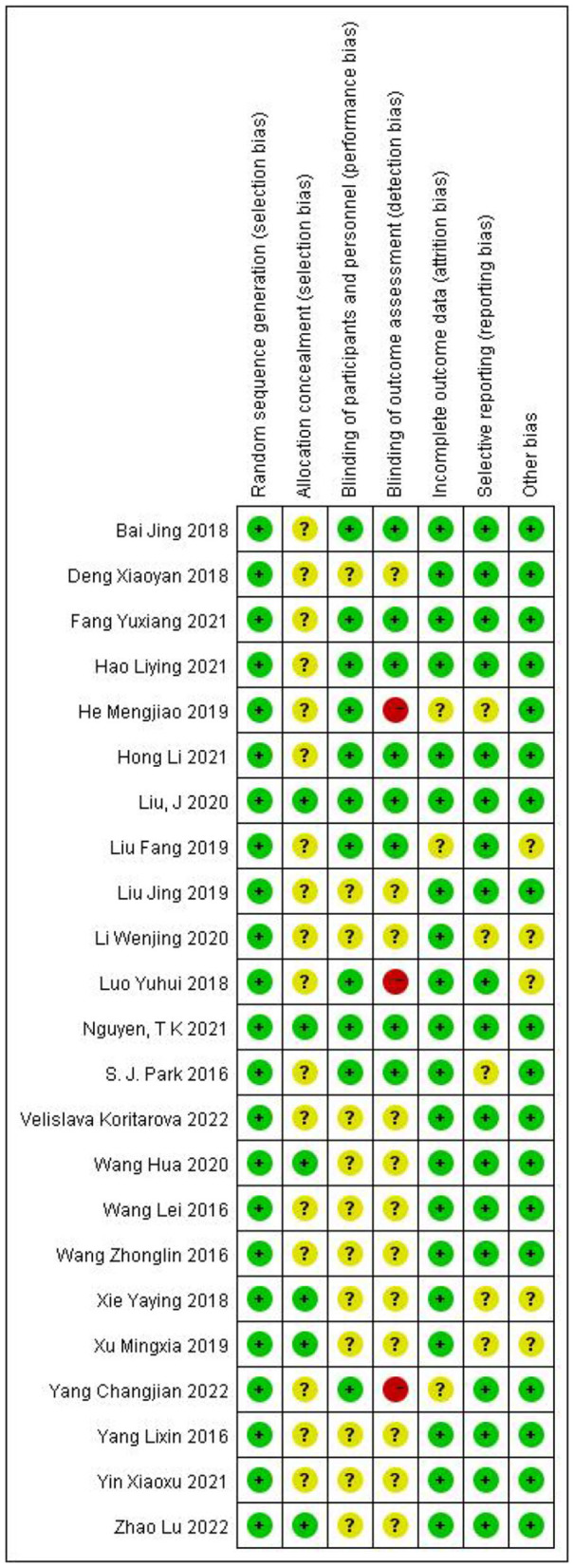
Risk of bias of included trials was assessed using the Cochrane risk of bias tool. Low risk = bias, if present, unlikely to significantly change results; unclear risk = bias raises some doubt about results; high risk = bias might significantly change results.

Of the 23 articles included in this meta-analysis, 1 described pediatric laparoscopic surgery, and 22 described laparoscopic surgery in adult patients, of whom 2 underwent gynecological laparoscopic surgery, and 3 described laparoscopic surgery in overweight patients. The summary data in Table 1 were processed in Review Manager (version 5.3) software to complete the data bias assessment. We assessed the certainty of the evidence using the GRADE approach, which is presented in Appendix 2. Figure 2 summarizes the details of the risk of bias assessment. Two trials were judged to be at low risk of bias, 18 were at unclear risk, and three were at high risk of bias. All trials produced appropriate random sequences, and nine trials reported appropriate allocation concealment.

**Figure 3:**
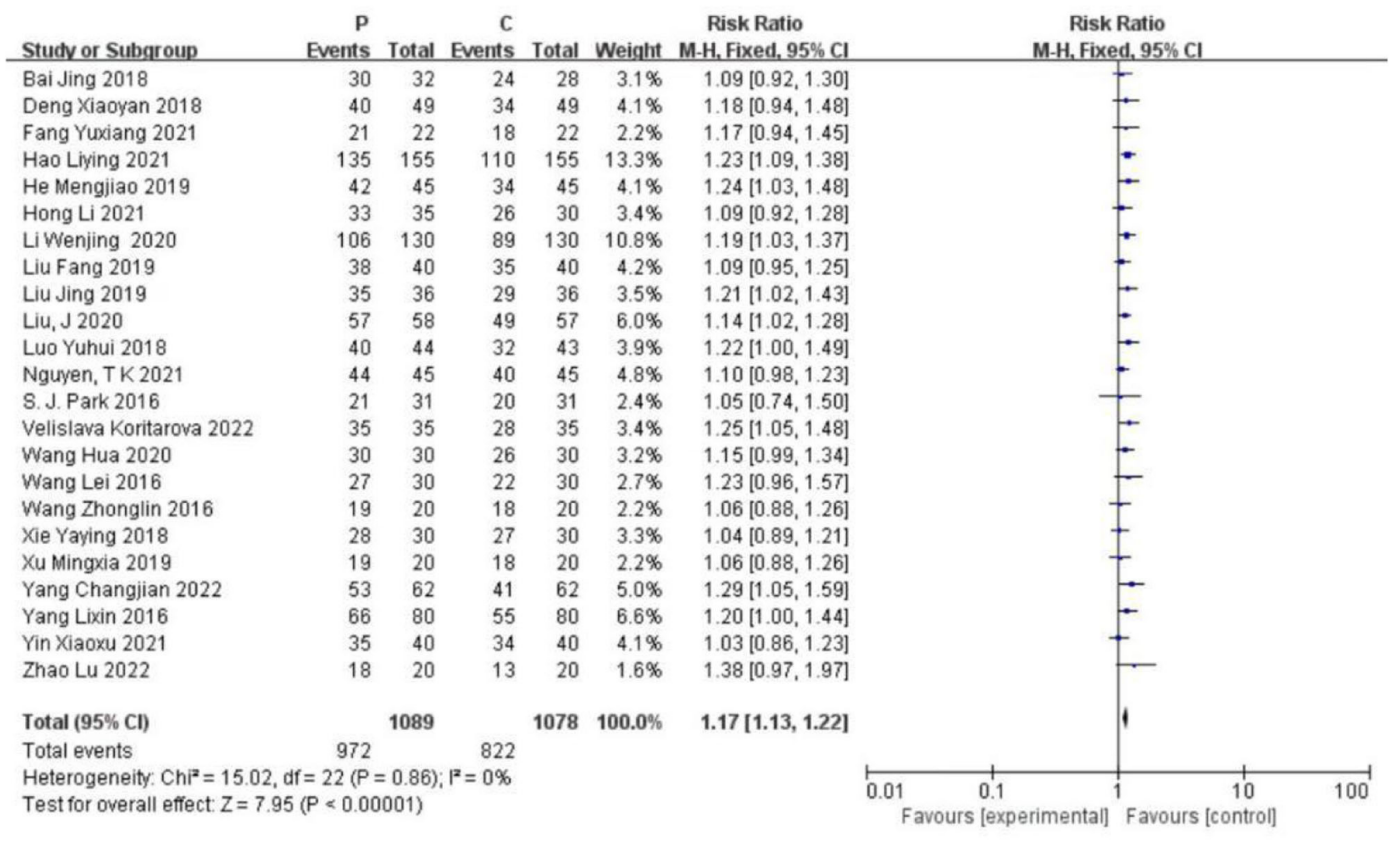
Effect of protective lung ventilation on pulmonary complications after laparoscopic surgery. A risk ratio >1.0 indicates a favorable effect on postoperative lung recovery. CI, confidence interval; event, number of people without pulmonary complications. *I*^2^ = 0%, P = 0.86.

After the heterogeneity test (*I*^2^ = 0% and *P* = 0.86 > 0.1), the Q test indicated that there was no heterogeneity between the selected literature in this study (the heterogeneity was not statistically significant), and the fixed effect was selected for pooled effect size. Twenty-three studies used a fixed-effect pooled RR = 1.17 (95% CI 1.13 to 1.22) and were statistically significant (Z = 7.95 and *P* = 0.00001 < 0.05), suggesting that protective lung ventilation is less likely to have pulmonary complications when applied to laparoscopic surgery than conventional lung ventilation and that protective lung ventilation is 1.17 times less like to cause complications than conventional lung ventilation. Figure 3 provides for details.

**Figure 4:**
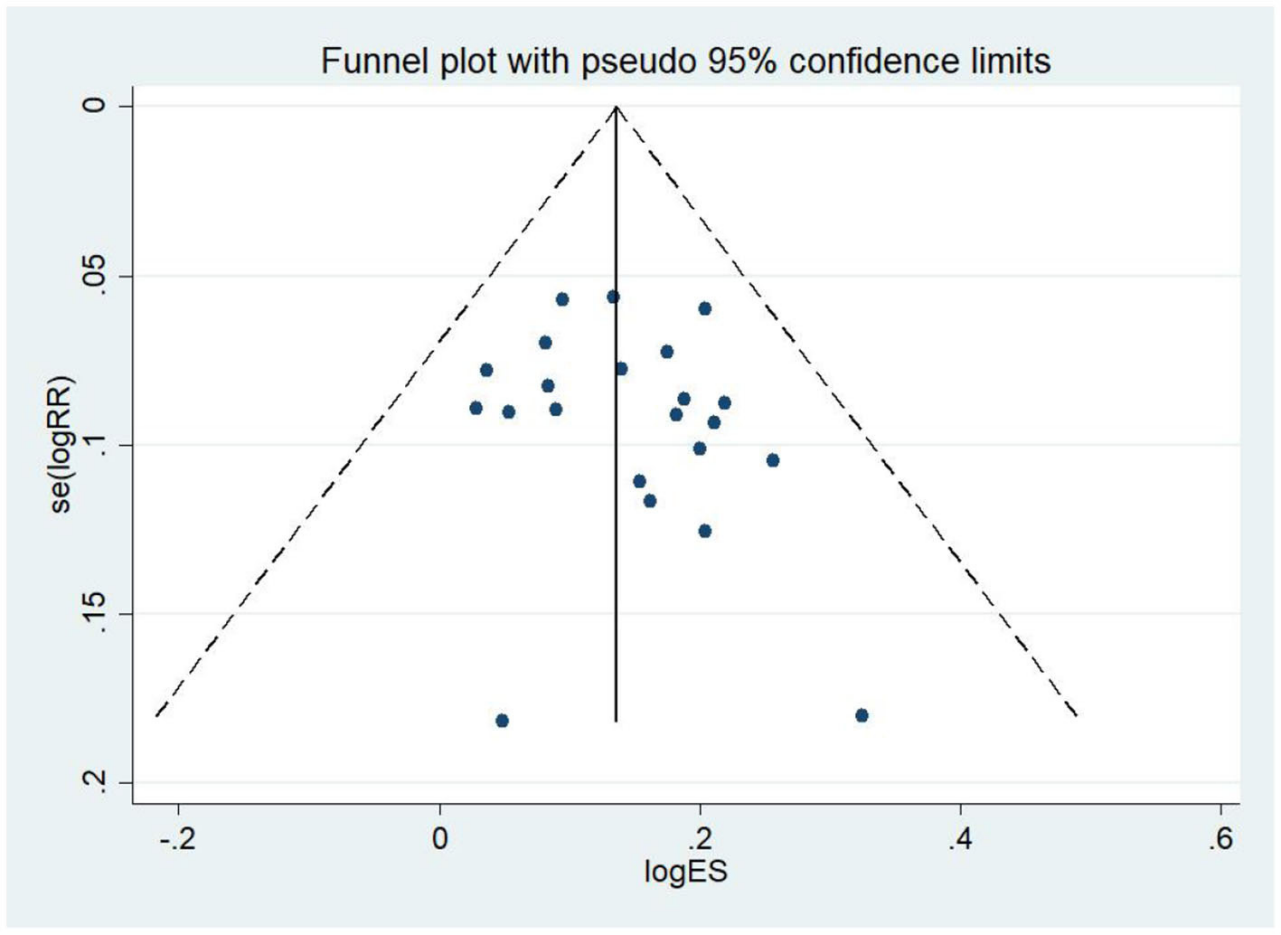
Test for bias (funnel plot). Preliminary judgement of bias was determined by whether it was symmetrical.

**Figure 5:**
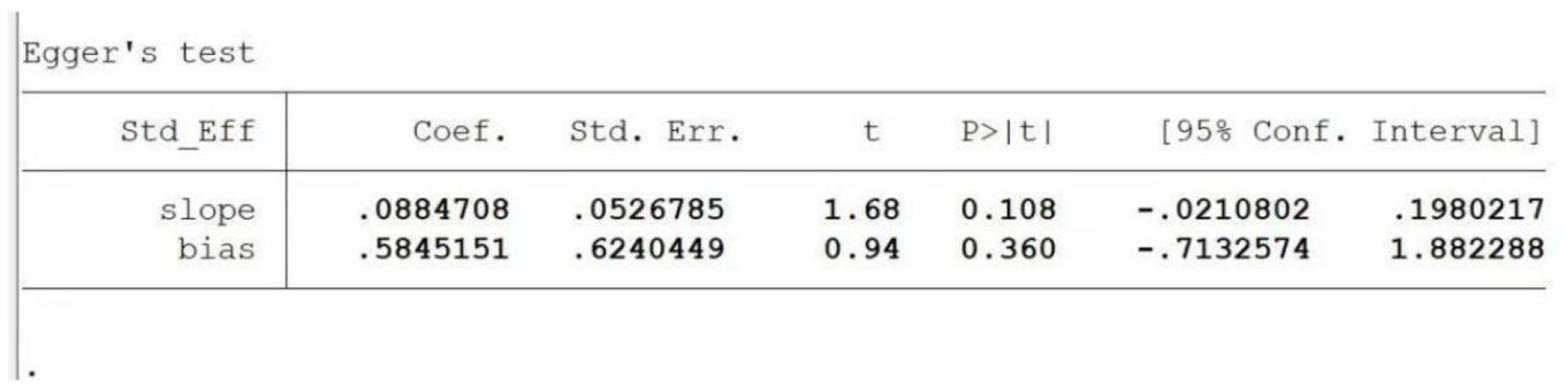
Protective lung ventilation test for bias of pulmonary complications after laparoscopic surgery. *P* > 0.1, the included data were unbiased and statistically significant.

By plotting funnel plots to investigate whether there was publication bias in the 23 articles of this study, visual findings showed that the funnel plots were symmetrical (Figure 4) and that there was no publication bias. The funnel plots were then evaluated in Stata software, version 14.0 to obtain *P* = 0.36 > 0.1, further confirming that the data were unbiased, and the conclusions of this study were accurate and reliable, as shown in Figure 5 with details.

**Figure 6:**
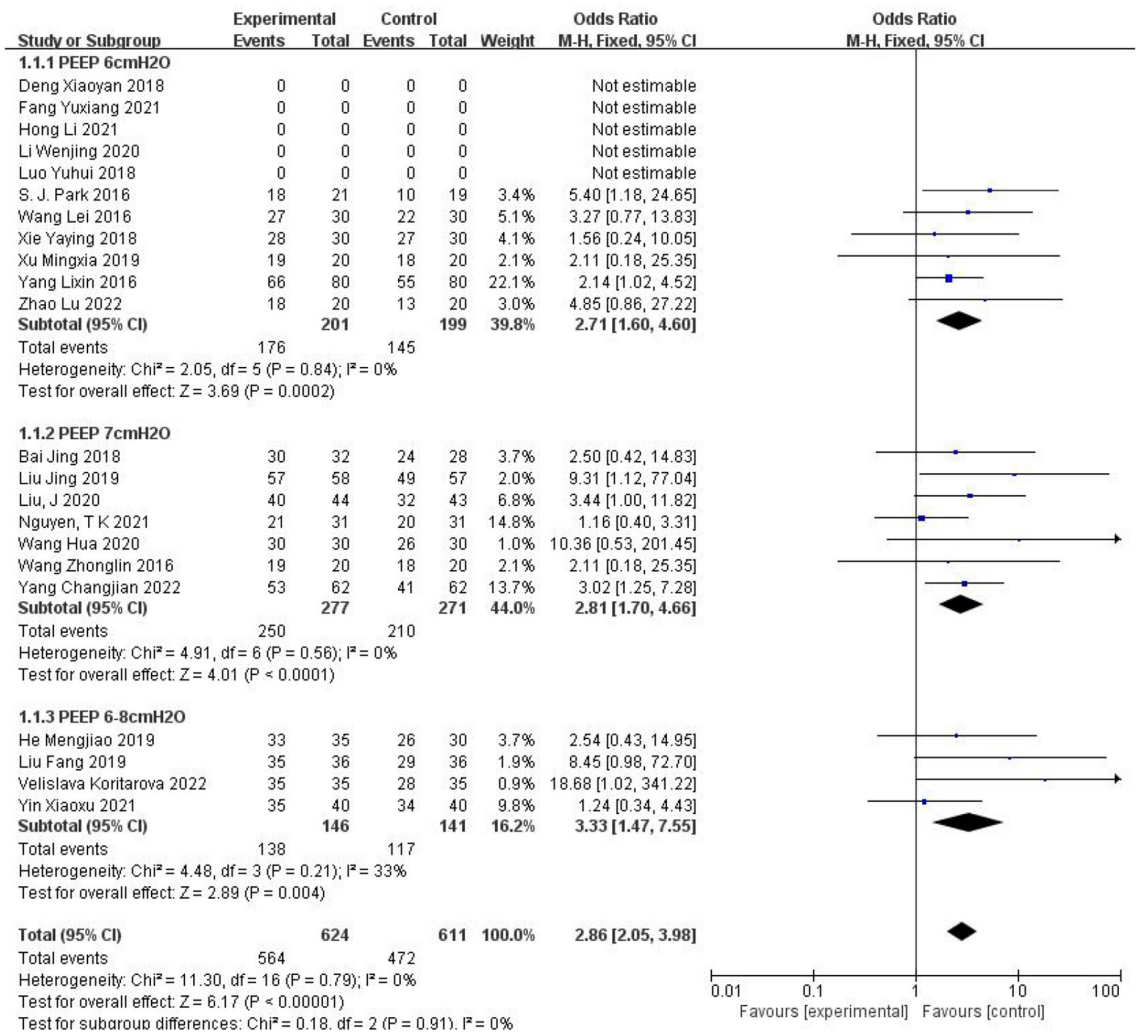
Subgroup analysis of different PEEP plus small tidal volume on the pulmonary complications. A risk ratio >1.0 indicates a favorable effect on postoperative lung recovery. CI, confidence interval; event, number of people without pulmonary complications.

Then, we further studied the subgroup analysis of the effects of different PEEP plus small tidal volume ventilation on pulmonary complications (Figure 6), and the results showed that when PEEP was in 6 cm H_2_O, RR = 2.71, *I*^2^ = 0%, *P* = 0.84, when PEEP was in 7 cm H_2_O, RR = 2.81, *I*^2^ = 0%, P = 0.56, there was no heterogeneity between the literature in the above two groups. We can conclude that different levels of PEEP plus small tidal volume ventilation reduce the incidence of pulmonary complications after laparoscopic surgery. The results of the between-group comparison were *I*^2^ = 0%, P = 0.91, which indicated that there was no heterogeneity between the groups.

We summarized specific pulmonary complications: atelectasis, hypoxemia, pneumonia, respiratory infections, as shown in Table 3. We found that the experimental groups with protective lung ventilation had significantly fewer PPCs than the control groups with conventional ventilation. Patients with PPCs are mainly characterized by atelectasis, hypoxemia, and pneumonia. And a few number of patients had respiratory infection, diffuse infiltrate, localized infiltrate, pleural effusion, increased thickness of interstitium, etc. Therefore, we can conclude that, when protective pulmonary ventilation is used in laparoscopic surgery, the probability of no pulmonary complications is 1.17 times that with conventional pulmonary ventilation, so laparoscopic surgery patients can have a better ventilation effect, with a reduced incidence of postoperative pulmonary complications, and protective ventilation can promote patient prognosis by adopting a protective lung ventilation strategy (small tidal volume + PEEP).

**Table 3:** Summary of specific pulmonary complications.

**Number**	**Atelectasis**		**Hypoxemia**		**Pneumonia**		**Respiratory infection**	
	**P group**	**C group**	**P group**	**C group**	**P group**	**C group**	**P group**	**C group**
1		2						
2		4					3	5
3		7						
4	24	41						
5	3	8				1		
6								
7		2					1	1
8								
9					1	7		
10								
11		4			2	1		
12								
13								
14	1	3	1	3				
15						4		
16		1	1	3				
17								
18	3	8	6	12				
19		1			1	1		
20		0	4	11	2	3		
21	2	1						
22	6	13	10	20	7	16		
23						1		
Total	39	95	22	49	13	34	4	6

## Discussion

For patients who require laparoscopic surgery under general anesthesia, there might not be a variety of serious lung diseases, but various factors, such as anesthetic drugs, mechanical ventilation, pneumoperitoneal pressure, special positioning, and surgical trauma used during surgery, can cause damage to the patient's lung tissue. Studies have found that when pneumoperitoneum pressure is at the level of 11–13 mm Hg it can lead to an average increase of 66% in atelectasis, greatly increasing the incidence of postoperative pulmonary complications.

Mechanical ventilation is one of the important conditions for the successful completion of laparoscopic surgery, but it can also cause ventilator-induced lung injury (VILI) while providing life support to patients. In the past, it was believed that the length of mechanical ventilation was directly proportional to the incidence of pulmonary complications. However, the latest research shows that even a short period of mechanical ventilation can cause VIIL and even ARDS in healthy lungs.

Protective lung ventilation strategies include small tidal volume ventilation and continuous positive airway pressure (PEEP). Relevant studies have shown that excessive tidal volume, blood transfusion, infection, and extracorporeal bypass during mechanical ventilation can lead to damage to healthy lungs (32). In animal experiments, it was found that, if healthy animals were subjected to mechanical ventilation with a large tidal volume for several hours, it caused deformation, necrosis, and exfoliation of alveolar epithelial cells and vascular endothelial cells, increased the level of inflammatory mediators in bronchoalveolar lavage, and significantly increased the expression of various cytokines (such as TNF). In this study, it was found that the use of small tidal volume protective pulmonary ventilation during mechanical ventilation could indeed reduce the incidence of pulmonary complications (33, 34), providing definitive evidence for clinical work.

Continuous positive airway pressure (PEEP) and the use of appropriate PEEP during mechanical ventilation can assist in collapsed alveolar remanoeuvres (35). Studies have shown that alveolar remanation can increase the functional residual gas volume and lung compliance of the lungs from a physiological point of view, improve the ventilation status and oxygenation status of patients, and reduce functional shunts in the lungs. The study found that, comparing small tidal volumes plus lower level PEEP and low tidal volumes plus high level PEEP, the former had a relatively large area of alveolar collapse and atelectasis during surgery and basically no tensile lung tissue. In the latter, there was hypertense lung tissue (36). Therefore, although small tidal volumes plus low-level PEEP cannot adequately maintain alveolar remanoestasis, it will not cause alveolar hypertension. However, small tidal volumes plus high-level PEEP can satisfactorily achieve the purpose of alveolar remanoeuvres, but at the same time, there is alveolar hypertonic damage, which has an impact on circulatory function. Therefore, too low or too high a level of PEEP has certain adverse effects. Choosing an appropriate PEEP is particularly important for mechanical ventilation and preventing postoperative pulmonary complications.

Protective lung ventilation strategies have received a lot of attention in recent years as a new approach to mechanical ventilation-related lung injury. Many studies have shown that protective lung ventilation has high clinical value for the treatment of patients with acute respiratory lung injury (ALI) and acute respiratory distress syndrome (ARDS), while there is a lack of clear clinical evidence in relevant studies in patients with better physical condition. In this study, we concluded that the use of protective lung ventilation strategies in general patients can effectively reduce the incidence of postoperative pulmonary complications by including patients of different ages and physical conditions. This demonstrates that the protective ventilation strategy with a small tidal volume plus moderate PEEP is also suitable for mechanical ventilation in general patients. The evidence shows that a target tidal volume of 6 mL/kg causes mild hypercapnia in patients with relatively normal lung function and gas exchange. Studies have shown that mild hypercapnia is permissible during ventilation because respiratory acidosis due to hypercapnia can increase respiratory motility, although this is based on the absence of craniocerebral lesions or cardiovascular diseases. In addition, in this study, we also analyzed the effect of small tidal volume plus different levels of PEEP on postoperative pulmonary complications in patients. Through subgroup analysis, we found that when PEEP was set to 6 or 7 cm H_2_O, compared with conventional ventilation group, it can effectively reduce the occurrence of postoperative pulmonary complications in patients, which provides the effective evidence for subsequent clinical work.

In summary, a protective lung ventilation strategy with a small tidal volume plus moderate levels of PEEP can be used to minimize ventilator-associated lung injury when mechanical ventilation is performed during laparoscopic surgery (3). Postoperative pulmonary complications, including atelectasis, pneumonia, and lung injury, are the most common complications and the main causes of morbidity and mortality, affecting the prognosis and prolonging the hospital stay. Therefore, we recommend the use of protective lung ventilation strategies during mechanical ventilation, which can effectively reduce the incidences of lung injury and lung infection. A strategy of low tidal volume + moderate positive end-expiratory airway pressure reduces the risk of lung injury and infection. In addition, the occurrence of VILI is also related to various factors, such as inspired oxygen concentration, ventilation mode, and pulmonary recruitment maneuvers, so we still require further research to optimize the protective lung ventilation strategy by adjusting the inspired oxygen concentration, improving the ventilation mode, and selecting a reasonable lung recruitment method.

## Limitations

This study has certain limitations. Firstly, in the process of screening the literatures, two people completed the process separately and summarized them, and there was a degree of subjectivity. Secondly, the 23 articles included patients of different ages, including children, adults, and the elderly, and the outcome indicators were inevitably affected by age, physical condition, lung function and other factors, which had an impact in our conclusion that the protective lung ventilation strategy used in laparoscopic surgery can effectively reduce the incidence of PPCs. In addition, we used pulmonary complications as an independent and complete indicator to demonstrate that protective lung ventilation strategies used in laparoscopic surgery are effective in reducing the incidence of PPCs after surgery. However, there was no detailed comparison of interventions for pneumonia, atelectasis, etc.

## Conclusion

Compared with conventional mechanical ventilation, protective lung ventilation reduces the incidence of postoperative pulmonary complications. For patients undergoing laparoscopic surgery, we suggest the use of protective lung ventilation, which is effective in reducing the incidence of lung injury and pulmonary infection. Implementation of a low tidal volume plus moderate positive end-expiratory pressure strategy reduces the risk of postoperative pulmonary complications. Postoperative pulmonary complications, including atelectasis, pneumonia, and lung injury, are the most common complications and the main causes of morbidity and mortality, affecting the prognosis and prolonging the hospital stay. Therefore, the use of protective lung ventilation strategy can facilitate the patient's recovery more quickly.

## Data availability statement

The original contributions presented in the study are included in the article/Supplementary material, further inquiries can be directed to the corresponding author.

## Author contributions

YW and LJ proposed and designed this study. MS and BY retrieved and selected the data and responsible for the extraction of the data and the quality assessment of all study data. MS performed a statistical analysis and summarized the data and drafted the manuscript. YW, BY, and TW revised it. All authors contributed to the article and approved the submitted version.
