# Supplementary material for: Effect of protective lung ventilation on pulmonary complications after laparoscopic surgery: a meta-analysis of randomized controlled trials
Source: Front Med (Lausanne). 2023 May 25;10:1171760. doi: 10.3389/fmed.2023.1171760 (PMC10248173; doi:10.3389/fmed.2023.1171760)
Supplement: Supplementary file 2 [file Data_Sheet_2.docx]

Supplementary Material

**Effect of protective lung ventilation on pulmonary complications after laparoscopic surgery: A meta-analysis**

**of randomized controlled trials**

First Author*, Menglin Sun^1^, Yuxia Wang^1*^, Bo Yang^1*^

*** Correspondence:** Yuxia Wang: [wyxwl@zzu.edu.cn;](mailto:wyxwl@zzu.edu.cn,) Bo Yang: yangboys@126.com

# Supplementary Figures and Tables

## Supplementary Tables

**Supplementary Table 1.** The search strategies of all search engines.

| **database** | **Search queries** |
| --- | --- |
| CNKI | (Subject: Laparoscopic (precise))OR (Subject: Laparoscopic surgery (precise)) OR (Subject: Porous Laparoscopy (precise))OR (subject: single-port flag-controlled microscopy (precise))OR(subject: transumbilical cavity Endoscopy (precise))OR (subject: transumbilical single-port laparoscopy (precise)) AND ((subject: lung Ventilation (precise)))OR (Patron: Protective Pulmonary Ventilation (precise))OR (Subject: Protective Ventilation (Precise))OR (Subject: Lung Protective Ventilation (Precise))OR (Subject: Lung Protective Ventilation (Precise)) OR (subject: lung-protective pathways (precise))OR (subject: lung-protective ventilation therapy (precise))OR (subject: lung-protective ventilation modes (precise)))OR (subject: lung-protective mechanical ventilation (Precise) ) ) AND ((Abstract: randomized controlled trials (precise))OR (Abstract: RCTs Experimental (precise))OR (Abstract: Randomized controlled (precise))OR (Abstract: RCT (precise))OR (Essentials: Randomized controlled studies (precise))) |
| CBM | (("RCT" [Common field: SMART] OR "Randomized controlled study" [Common field: SMART] OR "Randomized control" [Common field: Smart] OR "randomized" [Common field: Smart] OR "RCT" [Common field: Smart]) OR ("RCT" [Unweighted: Extended])) AND (("Protective Lung Ventilation" [Common Field: Smart] OR "Protective Ventilation" [Common Field: Smart] OR "Lung Protective Ventilation" [Common Field: Smart] OR "Lung Protective Ventilation" [Common Field: Smart] OR "Lung Protective Ventilation Strategy" [Common Field: Smart] OR "Lung Protective Ventilation Mode" [Common Field: Smart] OR "Lung-Protective Mechanical Ventilation" [Common Field: Smart]) OR (" Lung ventilation" [unweighted: extended])) AND (("Laparoscopic surgery" [Common field: intelligent] OR "Multi-hole laparoscopy" [Common field: intelligent] OR "Single port laparoscopy" [Common field: intelligent] OR "Transumbilical laparoscopy" [Common field: intelligent] OR "Transumbilical single-port laparoscopy" [Common field: intelligent]) OR ("Laparoscopy" [Unweighted: Extended])) |
| Wanfang | ( laparoscopic or laparoscopic surgery or multi-hole laparoscopy or single-port laparoscopy or transumbilical laparoscopy or transumbilical single-port laparoscopy ) and subject:( Lung ventilation or Protective lung ventilation or Protective ventilation or Lung protective ventilation or Lung protective ventilation or Lung protective ventilation or Lungprotective ventilation or Lung protective ventilation mode or Lung protective mechanical ventilation ) and subject: ( RCTs or RCTs or RCTs or RCTs) |
| PubMed | ((("Laparoscopes"[Mesh]) OR ((((((((Peritoneoscope[Title/Abstract]) OR (Celioscope[Title/Abstract])) OR (Laparoscope[Title/Abstract])) OR (Laparoscopic surgery[Title/Abstract])) OR (Porous laparoscopy[Title/Abstract])) OR (Single-port laparoscopy[Title/Abstract])) OR (Transumbilical laparoscopy[Title/Abstract])) OR (Transumbilical single-port laparoscopy[Title/Abstract]))) AND (("Pulmonary Ventilation"[Mesh]) OR (((((((((((Ventilation, Pulmonary[Title/Abstract]) OR (Airflow, Respiratory[Title/Abstract])) OR (Airflow, Expiratory[Title/Abstract])) OR (Protective pulmonary ventilation[Title/Abstract])) OR (Protective ventilation[Title/Abstract])) OR (Pulmonary protective ventilation[Title/Abstract])) OR (Lung protective ventilation[Title/Abstract])) OR (Lung protective strategies[Title/Abstract])) OR (Lung-protective ventilation therapy[Title/Abstract])) OR (Pulmonary protective ventilation mode[Title/Abstract])) OR (Lung-protective mechanical ventilation[Title/Abstract])))) AND (randomized controlled trial[Publication Type] OR randomized[Title/Abstract] OR placebo[Title/Abstract]) |
| Web of science | (Laparoscopes OR Peritoneoscope OR Celioscope OR Laparoscope OR Laparoscopic surgery OR Porous laparoscopy OR Single-port laparoscopy OR Transumbilical laparoscopy OR Transumbilical single-port laparoscopy ) AND (Pulmonary Ventilation OR Ventilation, Pulmonary OR Airflow, Respiratory OR Airflow, Expiratory OR Protective pulmonary ventilation OR Protective ventilation OR Pulmonary protective ventilation OR Lung protective ventilation OR Lung protective strategies OR Lung-protective ventilation therapyORPulmonary protective ventilation modeORLung-protective mechanical ventilation) AND (randomized controlled trial OR randomized OR placebo OR RCT) |
| Cochrane | (( Peritoneoscope):ab,ti,kw OR ( Celioscope):ab,ti,kw OR ( Laparoscope):ab,ti,kw OR ( Laparoscopic surgery):ab,ti,kw OR ( Porous laparoscopy):ab,ti,kw OR ( Single-port laparoscopy):ab,ti,kw OR ( Transumbilical laparoscopy):ab,ti,kw OR ( Transumbilical single-port laparoscopy):ab,ti,kw ) AND(( Ventilation, Pulmonary):ab,ti,kw OR ( Airflow, Respiratory):ab,ti,kw OR ( Airflow, Expiratory):ab,ti,kw OR ( Protective pulmonary ventilation):ab,ti,kw OR ( Protective ventilation):ab,ti,kw OR ( Pulmonary protective ventilation):ab,ti,kw OR ( Lung protective ventilation):ab,ti,kw OR ( Lung protective strategies):ab,ti,kw OR ( Lung-protective ventilation therapy):ab,ti,kw OR ( Pulmonary protective ventilation mode):ab,ti,kw OR ( Lung-protective mechanical ventilation):ab,ti,kw )AND(( randomized controlled trial):ab,ti,kw OR ( randomized):ab,ti,kw OR ( placebo):ab,ti,kw OR ( RCT):ab,ti,kw) |

## Supplementary Figures


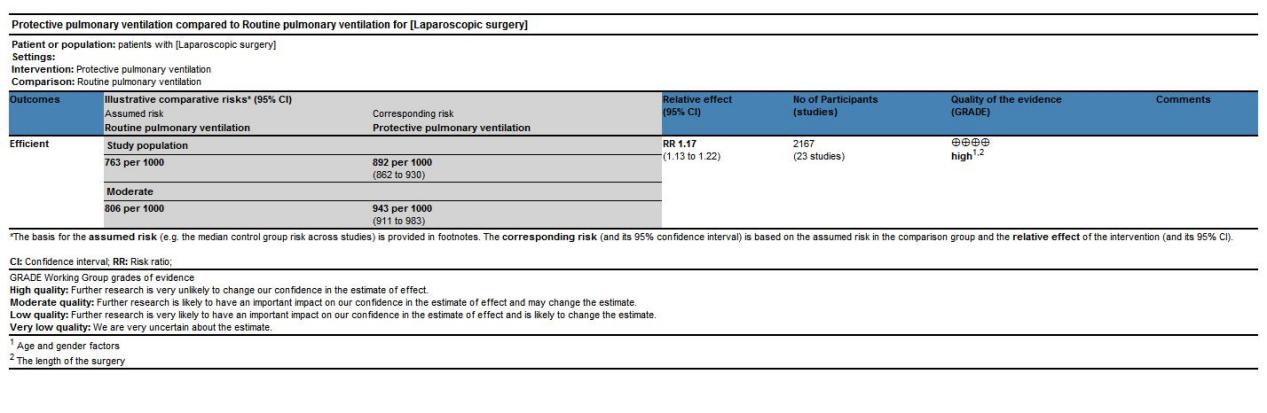


**Supplementary Figure 1:** The evidence quality assessment figure for 23 studies.


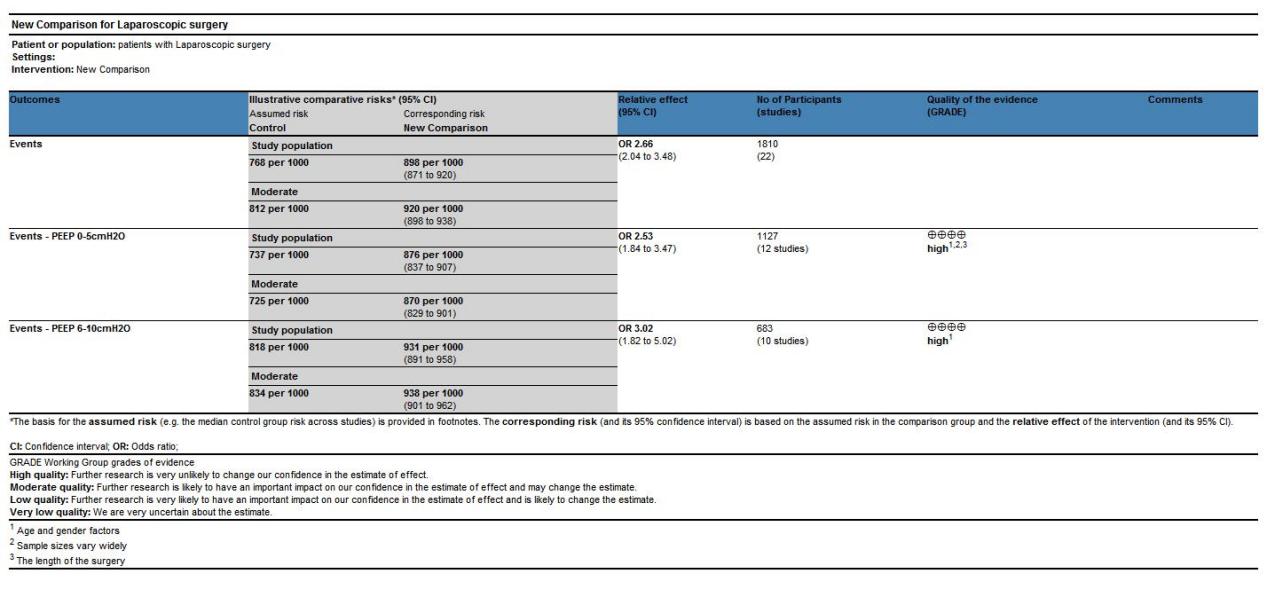


**Supplementary Figure 2:** The evidence quality assessment figure for 2 subgroups.
